# Impact of the CTSA on nutrition research infrastructure: Perspectives from research dietitian nutritionists

**DOI:** 10.1017/cts.2024.563

**Published:** 2024-11-06

**Authors:** Rachelle Bross, Catherine A. Chenard, Andrea Moosreiner, Amy Schweitzer

**Affiliations:** 1 Lundquist Institute for Biomedical Innovation at Harbor UCLA Medical Center, Torrance, CA, USA; 2 University of Iowa, Iowa City, IA, USA; 3 Medical College of Wisconsin, Milwaukee, WI, USA; 4 University of the District of Columbia, Washington, DC, USA

## Introduction

**Figure 1.**
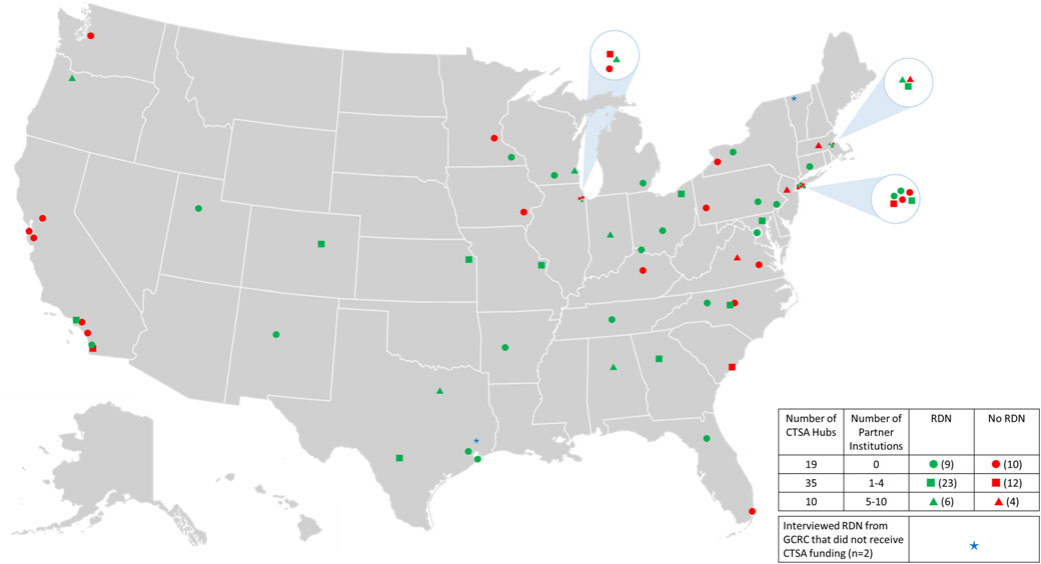
Clinical and Translational Science Award (CTSA) Program hubs: presence of Registered Dietitian Nutritionists (RDNs). To determine the proportion of Clinical and Translational Science Awards (CTSAs) with Registered Dietitian Nutritionists (RDNs), a list of the 64 CTSA awardees for fiscal year 22 was downloaded from https://ncats.nih.gov/files/CTSA_Partner_List_FY22-CTSA_Hub_Awards.pdf on June 26, 2022. Among this list are CTSA hubs with no partner institutions (circles), 1–4 partner institutions (squares), and 5–10 partner institutions (triangles). The list was cross-referenced with the National Association for Research Nutrition’s (NARN’s) membership list to identify sites with research RDNs (green symbols indicate the presence of RDN, red symbols indicate the absence of RDN). Websites for the remaining sites were reviewed for information on nutrition units and/or RDNs. When websites did not include this information, the CTSA was contacted by email and/or telephone to determine if the site had a research RDN. Of the 64 sites, 38 (59%) had research RDNs. The blue stars represent former General Clinical Research Centers (GCRCs) that are not CTSA sites and are now funded by their local institutions.

In 1959, the National Institutes of Health (NIH) established the General Clinical Research Center (GCRC) program for patient-focused, bench-to-bedside studies [1]. GCRCs provided infrastructure (beds, metabolic kitchens) and research staff (nutritionists, nurses, etc.) [2–5], so NIH-funded investigators could utilize these services at no cost [1,3]. Registered Dietitian Nutritionists (RDNs) trained in research were staffed at all centers. They provided expertise in controlled feeding studies, body composition, energy expenditure, and nutritional assessment. This expertise was standardized across GCRCs in part due to an organization of research nutritionists currently known as the National Association for Research Nutrition (NARN; https://www.researchnutrition.org). Funding for the approximately 77 GCRCs began to phase out in 2006 as the research emphasis transitioned to institution-wide training, education, and collaboration [6] under the Clinical and Translational Science Award (CTSA). GCRC units were included as part of CTSA applications; however, starting in 2014, the grant no longer allowed funding for their infrastructure/staff, including RDNs, metabolic kitchens, and nursing/laboratory services [7]. Financing was obtained from various cost recovery models [8] and institutional support but did not fully compensate for the loss of funding. Consequently, in 2022, only 38 (59%) of 64 CTSA hubs employed RDNs within nutrition research units (Figure 1).

Nathan and Nathan [2] lamented the demise of GCRCs, saying the loss would “deeply damage clinical research and demoralize the clinical research community.” To explore this perception through the experience of RDNs, NARN hosted panel discussions via Zoom in 2020 open to all 54 members, representing half of CTSA sites with RDNs. Twelve current and two former members responded to questions about their experiences during the GCRC to CTSA transition. To summarize their perspectives, responses were classified using principles of thematic analysis [9]. Results were organized according to domains and salient quotes in Table 1. This paper describes those themes and the impact of the change from GCRC to CTSA on research RDNs and their nutrition units.

**Table 1.** Thematic analysis of nutrition research experiences during CTSA transitions

**Question 1:** Compare your Nutrition Research department as a GCRC before the transition to a CTSA with what it is like now. Question 2: How has your role changed as a result of the transition from a GCRC to CTSA? Question 3: What limitations or challenges have you encountered? Question 4: What benefits or positive effects have you seen?	
**Domain: Definition**	**Supporting Quotes**
**Staffing:** Change in number and type of Nutrition Research staff.	“The loss of skilled people, the loss of a lot of research dietitians who have so much knowledge.” “when we were first funded, we had nine FTEs^a^ in our Bionutrition^b^ Department…now we have 1.5 FTEs.”
**Facilities (equipment/space):** Nutrition Research space and equipment (metabolic kitchens, dining rooms, body composition labs, storage, office space, and software).	“not having a kitchen and not having access to a kitchen to get food that we need for a study easily…it’s challenging doing feeding studies when you don’t have the facilities and the staff to do it.” “We did lose our inpatient unit.” “Space and equipment are the same as GCRC and CTSA…”
**Finances:** Fee charged for services provided, implementation of cost-recovery systems, and the challenges with funding and cost to conduct nutrition research studies.	“We are 100 percent fee for service now. But we do also receive institutional support. So we are not making back 100% of our budget by any means whatsoever.”
**Institutional Support:** Advocacy and financial support for Nutrition Research departments by CTSA and/or hospital/university administration.	“for the next seven years, I was threatened to close the kitchen because we were not making enough money to cover costs because there was no funding to cover the operations. CTSA had now changed its mission from providing services to providing guidance…” “the CTRC^c^ manager has been a huge champion for nutrition. I can really credit her for the reason why we’re still here, that she recognized that we have unique services that aren’t available anywhere else on campus…”
**Organization/Management:** Changes in management, collaborations, and relationships both within the Nutrition Research department and between other organizational components.	“We’re now part of a much larger organization, I’m able to network, interact and work with a much broader and diverse group of individuals.”
**Outlook:** Future projection of nutrition research professions, roles, and services.	“fear that we could be downsized or even closed if we cannot become cost neutral.” “I have a really good feeling about our unit. We’ve got so much support and we’ve got a lot of new types of studies coming in.”
**Role:** Scope of responsibilities and activities of research RDNs both within Nutrition Research departments and their CTSA.	“My time seems to be spread more thinly across many different activities, including workforce development, community engagement, as well as what I do in terms of being a research nutritionist.” “Now there’s a little bit more involvement with the administrators, like the business financial side of things…take on roles of figuring out cost recovery, marketing our services…”
**Services:** Scope and quality of services provided by Nutrition Research departments and utilization of services.	“[investigators having to pay 100% for services] dramatically reduces the number of studies that we’re involved with and the scope of any nutrition-related activities that would have been part of those studies.” “We’ve really shifted away from long-term feeding studies and now we do a lot more nutrition assessment, 24-hour recalls, food frequency questionnaires, diet records. And then the feeding that we do is usually really short-term for inpatients or a couple of meals for outpatients.” “we cut a lot of corners now because we’re limited on how much time we can spend on things.” “We have actually more services that are under our umbrella in the Bionutrition area than it was before.”
**Efficiency:** A ratio of input to output where input includes nutrition research staffing, finances, equipment, and space/location. Output includes quantity and quality of services and research support.	“We have done at least once or twice yearly process improvement projects for the last eight years, which has changed how we do diet production, really decreased the amount of time diet production takes, which has increased the scope of our practice…” “one dietitian aside from myself and we’re still to a certain extent offering a full range of services.”
**Professional Development:** Networking and knowledge exchange among RDNs and training and educating nutrition students.	“another big limitation. Old NAB [GCRC National Association of Bionutritionists]… speakers and annual meetings and the support that we had to meet with each other face-to-face once a year…” “We used to have the Masters nutrition… students rotate through …and we just could not support that anymore because we just didn’t have the time, and we also didn’t really have the studies.”

a FTE: Full Time Equivalent.

b Bionutrition/Bionutritionist: an alternate term used to describe research Dietitian/Nutritionist.

c CTRC: Clinical and Translational Research Center.

## Research dietitian nutritionist perspectives

Significant changes in financial policies under the CTSA impacted how or if RDNs provided nutrition services for investigators. One RDN commented, “We’re in the midst of a pendulum shift towards very little support for the kind of work that we have all been trained to do.” In addition to varying levels of institutional support, centers moved to cost recovery models where investigators paid for nutrition services previously provided at no cost. Nutrition staff salary costs were not fully recovered, leading to RDN and nutrition support staff reductions and, in some cases, closure of nutrition units. Facility changes varied, including loss of metabolic kitchens and location changes. Reported benefits included new kitchens, offices, equipment, and networking opportunities. Two RDNs described “champions” among their administration who advocated for continued nutrition research resources.

NARN members navigated the transition with flexibility and agility. Some expanded their role to include administrative tasks associated with a fee-for-service model, marketing their services, or working with the Community Engagement Program. Others began offering services such as exercise testing.

Due to funding cuts, some RDNs used convenience foods instead of whole foods for feeding studies, and when metabolic kitchens were eliminated, food was often procured from affiliated hospital kitchens. This reduced costs but sacrificed quality, accuracy, and reproducibility. Furthermore, RDNs assisted with food preparation when staffing reductions were necessary. When inpatient facilities were lost, RDNs supervised feeding studies at hotels and other locations.

Some RDNs expressed concerns, while others felt optimistic about the future and the institutional support for their services; “the opportunity is that we get to start over and do things a little bit different….” RDNs observed reduced requests for services, especially complex long-term feeding studies, which they attributed to the loss of inpatient facilities and investigator’s inability to cover the costs; “when what we did was free, everybody talked about…how important nutrition was…to public health challenges….” If disease prevention and treatment through nutrition research are not prioritized, the next generation of investigators may be unable to conduct complex nutrition studies. A quality concern noted was “outsourcing” of services such as dietary recalls and body composition measurements to untrained study coordinators to reduce costs. This outsourcing may further constrain nutrition staffing and has implications for the rigor and reproducibility of nutrition research.

RDNs were also concerned about the lack of time, funding, and studies needed to train the next generation of research RDNs. As one RDN considered retirement, she wondered, “Will they replace me with another dietitian, and will this dietitian have any research experience?”

Regret was expressed about the loss of the annual GCRC meeting where RDNs kept abreast of NIH research changes, shared best practices, and networked with peers. NARN helps fill this gap with webinars and member forums.

## Conclusion

For better and worse, the CTSA has changed the translational research process and forever altered how nutrition research units operate [10]. The financial constraints that resulted in the loss of RDNs and their expertise represent a “barrier for the efficacy of clinical and translational research, and consequently, the success of clinical and translational investigators” [11]. CTSA funding and cost recovery models are inadequate, in part because CTSA sites have varying degrees of institutional, private, and industry support. Furthermore, the R01 mechanism has been insufficient to equitably maintain nutrition research infrastructure across sites [12]. A comprehensive study to determine the type of nutrition research resources needed at all CTSA sites is critical. These resources should be provided through a modified CTSA grant mechanism that includes RDN salary support. Furthermore, NARN can provide nutrition research consultations and standardized research tools to the broader research community.

This perspective paper is the first to describe significant challenges NARN members face based on the experience of a subset of RDNs. RDNs have the unique skills to support the goals of the *2020-2030 Strategic Plan for NIH Nutrition Research* [13] and remain an integral part of accelerating discovery, promoting health, and training the next generation of researchers. As research priorities and NIH policies evolve, RDNs will continue to advance translational science and adapt to new challenges and opportunities.
